# Serum neurofilament light chain but not serum glial fibrillary acidic protein is a marker of early Huntington’s disease

**DOI:** 10.1007/s00415-025-12901-y

**Published:** 2025-02-01

**Authors:** Beatrice Heim, Elias Mandler, Marina Peball, Federico Carbone, Katarína Schwarzová, Rina Demjaha, Cansu Tafrali, Arabella Buchmann, Michael Khalil, Atbin Djamshidian, Klaus Seppi

**Affiliations:** 1 Department of Neurology, Medical University of Innsbruck, Innsbruck, Austria; 2 Department of Neurology, Medical University of Graz, Graz, Austria; 3 Department of Neurology, Hospital Kufstein, Kufstein, Austria

**Keywords:** Huntington’s disease, Mutation carriers, Biomarkers, Glial fibrillary acidic protein, Neurofilament light, Olfactory dysfunction, Neurocognition

## Abstract

**Background:**

Huntington’s disease (HD) is caused by CAG trinucleotide expansion on chromosome 4, leading to mutant Huntingtin production. Premanifest carriers show no obvious clinical signs, and early symptoms progress slowly. Fluid biomarkers like neurofilament light (NfL) and glial fibrillary acidic protein (GFAP), measurable in cerebrospinal fluid and serum (sNfL, sGFAP), offer potential predicting HD progression.

**Objective:**

To assess the role of sGFAP and sNfL and clinical biomarkers in different disease stages and correlate with disease progression.

**Methods:**

HD mutation carriers were categorized into clinical stages according to their motor symptoms and functional capacities. The Unified HD Rating Scale, cognitive assessments and olfactory tests were used to characterize the patients clinically. Furthermore, sNfL and sGFAP levels were assessed.

**Results:**

We consecutively included 44 HD mutation carriers (13 premanifest HD (preHD), 18 in early (early HD) and 13 in advanced (advanced HD) disease stages) and 19 healthy controls (HC). Advanced HD patients performed worse on all clinical tasks and had higher sGFAP and sNfL levels compared to other groups (all p values < 0.05). We did not find difference in sGFAP levels between the preHD, early HD and HC group  (all p values > 0.05). In contrast, sNfL levels differed significantly between preHD and early HD, and HC (all p values < 0.05). ROC curve analysis revealed that the AUC of sGFAP (0.970) exhibited superior discriminatory accuracy compared to sNfL (0.791) levels in separating advanced from early HD patients. By contrast, ROC curve analysis revealed that the AUC of sNFL (0.988) exhibited superior discriminatory accuracy compared to sGFAP (0.609) levels in separating all HD mutation carriers from HC.

**Conclusions:**

Our study indicates that sNfL can detect changes in very early and premanifest HD stages, whereas sGFAP showed differences in more advanced stages only.

## Introduction

Huntington’s disease (HD) is a neurodegenerative disorder caused by CAG trinucleotides expansion on chromosome 4 resulting in the production of mutant Huntingtin (mHtt) [1].

In HD, premanifest mutation carriers do not exhibit obvious clinical signs, and even in patients in the early manifest stage, the progression of clinical symptoms is relatively slow and subtle. This slow progression can complicate the detection of significant clinical improvements in clinical trials, particularly those targeting premanifest individuals. Neuroimaging biomarkers offer the advantages of being non-invasive, standardized, and accessible. Nevertheless, neuroimaging techniques like MRI and PET-CT are costly and inconvenient, and the involuntary movements of patients can compromise image quality. Wet biomarkers derived mainly from cerebrospinal fluid (CSF) and blood can reflect disease pathogenic processes at the molecular level directly and might unravel the potential pathogenesis of HD.

One well established fluid biomarker is neurofilament light (NfL), which can be measured in CSF as well as in blood serum (sNfL) and plasma [2, 3]. While NfL demonstrates effective tracking of HD progression in CSF and blood, the need for additional biomarkers to comprehensively reflect this progression is evident.

Glial Fibrillary Acidic Protein (GFAP), an intermediate filament protein characterizing astrocyte activity, integrity and cellular activation. While the potential utility of serum GFAP (sGFAP) as a biomarker has been investigated across various neurological conditions, only little data is available on its specific role in the context of HD [4, 5].

The aim of this study was to better characterize the role of sGFAP as a body fluid biomarker in HD. To this end, we assessed sNfL, an established biomarker for neuro-axonal injury, alongside evaluating the astrocytic marker sGFAP and different clinical biomarker changes across various disease stages in HD patients.

## Methods

The study was approved by local ethics committee of the Medical University of Innsbruck, Austria, and all participants provided written informed consent (AN1979 336/4.19 401/5.10 (4464a)).

### Study participants

Participants were consecutively recruited during their routine follow up visit in a specialized outpatient clinic of the Medical University of Innsbruck, Department of Neurology, Austria. All HD patients and mutation carriers had genetically confirmed HD. PIN_HD_ scores were computed as described elsewhere [6]. For all HD patients, the CAG-Age-Product (CAP) score was calculated. All participants were clinically assessed by the same neurologists (B.H., K.S.) and symptoms rated using the Unified Huntington’s Disease Rating Scale (UHDRS).

We included only participants with a Mini Mental state Examination (MMSE) of ≥ 24 points, and without known circumstances possibly associated with olfactory dysfunction (e.g. chronic rhinosinusitis). Only participants without neurological conditions (except of HD) were included in the study.

Patients were categorized into premanifest HD patients (preHD) if the UHDRS total motor score (UHDRS-TMS) was ≤ 5. HD mutation carriers with the score of UHDRS-TMS > 5 were classified as manifest HD, who would be further separated into early or advanced disease stage according to their functional capacities (UHDRS total functional capacity, UHDRS-TFC) [7]: early HD: stage 1 (11 ≤ UHDRS-TFC ≤ 13) and stage 2 (7 ≤ UHDRS-TFC ≤ 10); late HD: stage 3 (3 ≤ UHDRS-TFC ≤ 6;) and stage 4 (1 ≤ UHDRS-TFC ≤ 2).

All biomarkers including fluid biomarkers, cognitive and olfactory testing were assessed after clinical evaluation including disease staging of the participants.

### Serum neurofilament light (sNfL) an serum glial fibrillary acidic protein (sGFAP)

Venous blood was collected from each participant. Serum was obtained after blood samples were centrifuged at 2000 × g for 10 min at 21 °C and then stored at − 80  °C.

sNfL was analysed with a commercial ultrasensitive single molecule array (Simoa) NfL-assay (reference number: 103186) on an SR-X platform (Quanterix) [8, 9]. Measurements were conducted in duplicate. Samples that could not be measured with a coefficient of variation < 20% were excluded from statistical analyses.

sGFAP levels were measured with a Single Molecule Array (Simoa®, GFAP single-plex discovery kit, Reference Number: 102336) on the HD-X platform according to the manufacturer’s instructions (Quanterix, Billerica, MA, USA). The assays’ lower limit of quantification lies at 0.686 pg/mL and the limit of detection lies at 0.211 pg/ mL. All measurements were above these limits. Samples that could not be measured with a coefficient of variation < 20% were excluded from statistical analyses.

### Neuropsychological and olfactory testing

Participants underwent a cognitive test battery including the MMSE, the trail making test part A (TMT A) and B (TMT B), as well as the Symbol Digit Modality Task (SDMT).

Olfactory functioning was assessed using the Sniffin’ Sticks 16-item identification and discrimination test (Burghart Medizintechnik, Wedel, Germany) [10, 11].

### Statistics

Statistical analyses were performed using SPSS 29.0. To test for normal distribution, the Kolmogorov–Smirnov test was used. Parametric and non-parametric tests were used for statistical analysis depending on the distribution and the scale type of variables. The significance level was set at two-sided p value of < 0.05 using a Bonferroni adjustment for group comparisons. Sum scores of the period of time to completion in the TMT part A and B were calculated per participant with a maximum of 240 s per task. sNfL and sGFAP were corrected for age, sex, body mass index (BMI), glomerular filtration rate (GFR), and CAG repeats. Neuropsychological test values and olfaction were corrected for age, sex, MMSE, and CAG repeats.

Group differences were assessed by ANCOVA with post hoc pairwise comparison. To explore the association of wet biomarkers with PIN_HD_, we performed a linear regression analysis with PIN_HD_ as dependent variable and sNFL and sGFAP as independent variables, correcting for sex, BMI and GFR. Because the calculation of PIN_HD_ includes age and CAG repeat length, we did not correct additionally for these variables. P-values of these linear regression analyses were adjusted using the False Discovery Rate (FDR) correction and their p-values are FDR-corrected.

ROC curve analyses were performed to investigate the ability of sNFL and sGFAP to distinguish controls from HD mutation carriers and early from advanced HD, respectively.

## Results

All demographic details and clinical results are summarized in Table 1.

**Table 1:** Demographic characteristics and test results

	preHD	Early HD	Advanced HD	HC	*p* value
Number (*n*)	13	18	13	19	
Male/female^a^	8/5	7/11	6/7	8/11	0.453
Age (years)^b^ (± SD)	43.39 ± 10.86	53.06 ± 12.57	54.00 ± 16.11	42.11 ± 12.74	preHD vs early HD *p* = 0.283 preHD vs advanced HD *p* = 0.260 preHD vs HC = 1.00 early HD vs advanced HD *p* = 1.00 early HD vs HC *p* = 0.082 advanced HD vs HC *p* = 0.087
BMI^b^ (± SD)	25.13 ± 3.93	21.41 ± 2.25	19.95 ± 2.58	23.42 ± 1.55	preHD vs early HD *p* = 0.008* preHD vs advanced HD *p* < 0.001*** preHD vs HC *p* = 0.702 early HD vs advanced HD *p* = 1.00 early HD vs HC *p* = 0.320 advanced HD vs HC *p* = 0.029*
MMSE^b^ (± SD)	29.54 ± 0.97	27.89 ± 2.19	26.67 ± 2.29	29.58 ± 0.84	preHD vs early HD *p* = 0.046* preHD vs advanced HD *p* < 0.001*** preHD vs HC *p* = 1.00 early HD vs advanced HD *p* = 0.439 early HD vs HC *p* = 0.016* advanced HD vs HC *p* < 0.001***
Educational years^b^ (± SD)	11.62 ± 2.44	10.90 ± 2.08	9.38 ± 1.78	11.05 ± 1.94	preHD vs early HD *p* = 1.00 preHD vs advanced HD *p* = 0.795 preHD vs HC *p* = 1.00 early HD vs advanced HD *p* = 1.00 early HD vs HC *p* = 1.00 advanced HD vs HC *p* = 1.00
UHDRS-TMS^b^ (± SD)	2.46 ± 2.37	20.50 ± 10.60	40.77 ± 17.63	n.a	preHD vs early HD *p* < 0.001*** preHD vs advanced HD *p* < 0.001*** early HD vs advanced HD *p* < 0.001***
UHDRS-TFC^b^ (± SD)	13.00 ± 0.00	10.72 ± 1.84	5.92 ± 2.53	n.a	preHD vs early HD *p* < 0.004** preHD vs advanced HD *p* < 0.001*** early HD vs advanced HD *p* < 0.001***
CAG repeats^b^ (± SD)	43.39 ± 2.06	44.00 ± 3.46	45.92 ± 8.68	n.a	preHD vs early HD *p* = 1.00 preHD vs advanced HD *p* = 0.692 early HD vs advanced HD *p* = 0.979
CAP Score^b^ (± SD)	410.83 ± 89.74	510.20 ± 68.47	557.13 ± 122.75	n.a	preHD vs early HD *p* 0.017* preHD vs advanced HD *p* < 0.001*** early HD vs advanced HD *p* = 0.524
PIN_HD_^b^ (± SD)	0.84 ± 0.97	2.60 ± 1.51	4.004 ± 1.68	n.a	preHD vs early HD *p* = 0.005** preHD vs advanced HD *p* < 0.001*** early HD vs advanced HD *p* = 0.026*
Glomerular filtration rate (ml/min/1,73 m^2^) (± SD)^##^	59.62 ± 0.96	59.06 ± 2.84	59.31 ± 1.18	59.53 ± 1.02	preHD vs early HD *p* = 0.807 preHD vs advanced HD *p* = 1.00 preHD vs HC *p* = 1.00 early HD vs advanced HD *p* = 1.00 early HD vs HC *p* = 1.00 advanced HD vs HC *p* = 1.00
sNfL (pg/mL)^b+^ (95% CI)^§^	21.32 (13.13, 29.51)	35.95 (29.00, 42.91)	50.40 (42.21, 58.59)	7.282 (6.70, 14.94)	preHD vs early HD *p* = 0.041* preHD vs advanced HD *p* < 0.001*** preHD vs HC *p* = 0.037* early HD vs advanced HD *p* < 0.001** early HD vs HC *p* < 0.001*** advanced HD vs HC *p* < 0.001***
sGFAP (pg/mL)^b+^ (95% CI)^§^	111.99 (77.66, 146.32)	148.13 (127.64, 170.08)	282.20 (257.88, 306.22)	136.08 (126.63, 167.52)	preHD vs early HD *p* = 0.701 preHD vs advanced HD *p* < 0.001*** preHD vs HC *p* = 0.524 early HD vs advanced HD *p* < 0.001*** early HD vs HC *p* = 1.00 advanced HD vs HC *p* < 0.001***
Correct odor identification^b^ (± SD)^§§^	11.00 ± 3.70	9.67 ± 3.48	8.62 ± 2.60	14.95 ± 0.91	preHD vs early HD *p* = 0.472 preHD vs advanced HD *p* = 0.035* preHD vs HC *p* < 0.001*** early HD vs advanced HD *p* = 0.116 early HD vs HC *p* < 0.001*** advanced HD vs HC *p* < 0.001***
Correct odor discrimination^b^ (± SD)^§§^	9.88 ± 2.36	8.67 ± 3.26	6.20 ± 2.95	14.95 ± 0.91	preHD vs early HD *p* = 0.718 preHD vs advanced HD *p* = 0.040* preHD vs HC *p* < 0.001*** early HD vs advanced HD *p* = 0.010* early HD vs HC *p* < 0.001*** advanced HD vs HC *p* < 0.001***
TMT-A^b^ (± SD)^§§^	30.46 ± 15.72	78.06 ± 68.27	107.33 ± 78.43	28.90 ± 9.24	preHD vs early HD *p* = 0.003** preHD vs advanced HD *p* 0.002** preHD vs HC = 1.00 early HD vs advanced HD *p* = 0.011* early HD vs HC *p* < 0.001*** advanced HD vs HC *p* < 0.001***
TMT-B^b^ (± SD)^§§^	72.15 ± 56.10	151.19 ± 83.24	201.33 ± 59.72	55.58 ± 12.62	preHD vs early HD *p* = 0.014** preHD vs advanced HD *p* = 0.010** preHD vs HC *p* = 0.066 early HD vs advanced HD *p* = 0.086 early HD vs HC *p* < 0.001*** advanced HD vs HC *p* < 0.001***
SDMT^b^ (± SD)^§§^	39.15 ± 12.24	31.06 ± 15.53	20.69 ± 8.71	46.95 ± 10.89	preHD vs early HD *p* = 0.883 preHD vs advanced HD *p* = 0.030* preHD vs HC *p* = 0.835 early HD vs advanced HD *p* = 0.108 early HD vs HC *p* = 0.047* advanced HD vs HC *p* = 0.035*

*BMI* body mass index, *GFAP* glial fibrillary acidic protein, *GFR* glomerular filtration rate, *HD* patients with manifest HD, *preHD* premotor HD mutation carriers, *HC* healthy controls, *MMSE* Mini Mental State Examination, *PIN*_*HD*_ normed version of the Prognostic Index for Huntington's Disease, *SD* standard deviation, *SDMT* Symbol Digit Modality Task, *sNfL* serum Neurofilament Light, *TMT A* Trail Making Test Part A, *TMT B* Trail Making Test Part B, *UHDRS* Unified Huntington’s Disease Rating Scale, *UHDRS-TMS* Unified Huntington’s Disease Rating Scale—Total Motor Score, *UHDRS-TFC* Unified Huntington’s Disease Rating Scale Total Functional Capacity

The significance level is set at *p < 0.05. **p < 0.05, ***p ≤ 0.001. P-values of post-hoc comparisons are adjusted by Bonferroni correction for multiple comparisons. ^+^ median with 95% CI. ^a^ Fisher’s exact. ^b^ Nonparametric tests (Mann–Whitney U test; Kruskal–Wallis one-way analysis of variance). ^#^ Disease staging: premanifest: UHDRS-TMS ≤ 5.; early HD: stage 1 (11 ≤ UHDRS-TFC ≤ 13) and stage 2 (7 ≤ UHDRS-TFC ≤ 10); advanced HD: stage 3 (3 ≤ UHDRS-TFC ≤ 6) and stage 4 (1 ≤ UHDRS-TFC ≤ 2). ^##^ The glomerular filtration rate was measured at a maximum of 60 ml/min/1,73 m^2^; values above this were not recorded. ^§^ corrected for age, BMI, GFR, sex; CAG repeats; ^§§^ corrected for age, sex, MMSE and CAG repeats

We consecutively included 31 manifest HD patients (18 early HD, 13 advanced HD), 13 premanifest HD mutation carriers (preHD), and 19 HC. There was no difference in age between the groups (all p values > 0.05) or sex (p = 0.453).

All HD mutation carriers showed significant worse performance in odor identification and discrimination compared to HC (all p values < 0.001). Furthermore, we found significant worse performance in all cognitive tasks comparing early and advanced HD patients with HC (all p values < 0.05), but we could not find any significant differences between HC and preHD participants (all p values > 0.05).

**Fig. 1:**
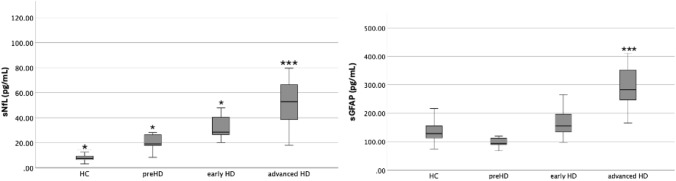
sNfL and sGFAP concentrations (pg/mL) per group. Boxplots of individual sNfL and sGFAP levels. *p < 0.05; **p < 0.01; ***p < 0.001. *advanced HD* HD patients in advanced disease stages (i.e. stage 3 and 4); early HD, HD patients in early disease stages (i.e. stage 1 and 2), *HC* healthy controls, *preHD* premanifest HD mutation carriers

sGFAP (see Table 1 and Fig. 1).

While the advanced HD group showed significant higher sGFAP levels compared to all other groups (all p values < 0.001), we did not find further group differences (all p values > 0.05).

Moreover, linear regression analysis demonstrated a significant association of sGFAP with PIN_HD_. (p = 0.044, ß = 0.391, FDR < 0.005).

All measurements were within the measurement range, i.e., above the lower limit of detection and below the upper limit of detection.

sNfL (see Table 1 and Fig. 1).

We found significant lower sNfL levels in the preHD group compared all other HD groups (all p-values < 0.05). Early HD patients had significant lower sNfL levels than advanced HD patients (p = 0.009), and HC showed lower sNfL levels than preHD (p < 0.001), early HD (p < 0.001) and advanced HD patients (p < 0.001). Furthermore, linear regression analysis revealed a significant association of sNFL with PIN_HD_ (p < 0.001, ß = 0.687, FDR < 0.005).

All measurements were within the measurement range, i.e., above the lower limit of detection and below the upper limit of detection.

**Fig. 2:**
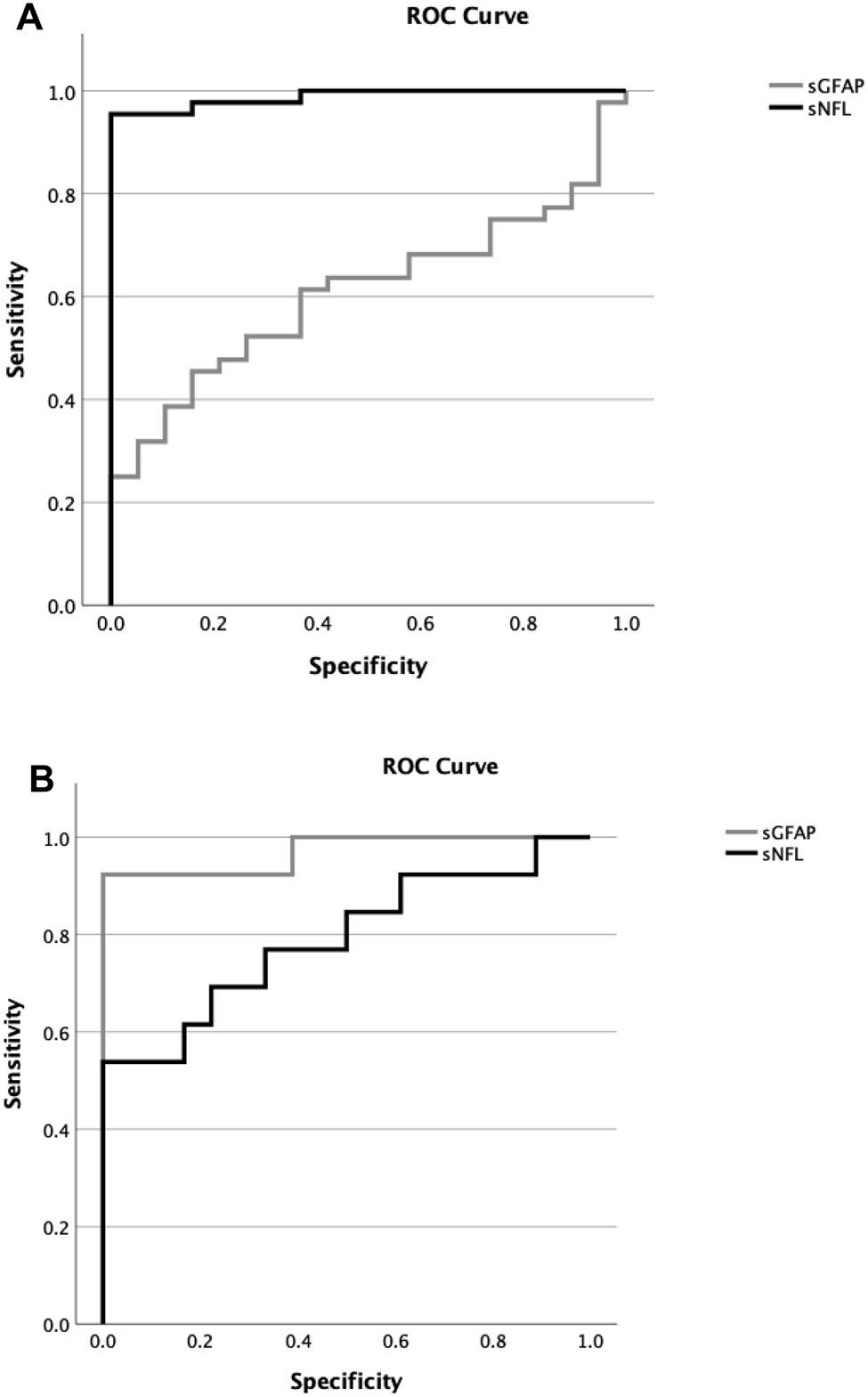
**A** Receiver operator characteristic curve of the sNFL and sGFAP in discriminating controls from all HD mutation carriers. **B** Receiver operator characteristic curve of the sNFL and sGFAP in discriminating advanced from early HD patients

ROC curve analyses (Fig. 2A, B).

ROC curve analysis revealed that the AUC of sNFL (0.988) exhibited superior discriminatory accuracy compared to sGFAP (0.609) levels in separating all HD mutation carriers from healthy controls (difference of AUCs 0.379, 95% CI 0.244–0.514, *p* < 0.001) (Fig. 2 A). By contrast, ROC curve analysis revealed that the AUC of sGFAP (0.970) exhibited superior discriminatory accuracy compared to sNfL (0.791) levels in separating advanced from early HD patients (difference of AUCs 0.179, 95% CI 0.002–0.357, *p* = 0.048) (Fig. 2 B).

## Discussion

Predicting the disease onset of HD in premanifest individuals is an unmet need and currently an area of active research.

With increasing exploration of interventions expected to elicit systemic effects in HD, the significance of blood-based biomarkers is becoming more prominent in the future [12]. It is crucial to correlate blood-based biomarkers with corresponding clinical biomarkers within the same cohort to evaluate their overall utility.

The aim of this single-centre study was to evaluate clinical and blood biomarkers in different stages of manifest HD patients and premanifest HD mutation carriers.

All participants underwent a clinical test battery including cognitive tasks, odor identification and discrimination, as well as blood-based biomarkers including sNfL and sGFAP.

As expected, we found that HD patients in advanced disease stages showed higher sGFAP and sNfL levels and performed worse on all clinical tasks compared to the other groups.

We did not find differences in sGFAP levels in early or premanifest disease stages compared with controls, but we found significant elevated levels in advanced disease stages.

Indeed, the ROC curve analysis confirmed the superiority of sGFAP in discriminating advanced from early HD patients compared to sNfL.

These results reflect the findings from recent studies, which found that plasma sGFAP is elevated at more advanced clinical stages in HD and could therefore possibly be regarded as a good marker for tracking disease progression in manifest stages [4, 13].

Nevertheless, in this study [4], no differences in sNfL levels between different HD disease stages (I-II versus III-IV) were found, whereas in our study, we could show significant differences in sNfL levels between early and advanced disease stages, as well as between premanifest HD patients and HC, and found a correlation with progression markers.

Our findings are in line with further studies, which found blood GFAP levels to be a marker of disease progression [4] and demonstrated that CSF and plasma NfL were elevated in a preHD cohort approximately 24 years from predicted clinical onset [14]. One study [15] assessed the longitudinal trajectory of NfL levels in HD patients over a 5-year period, suggesting that plasma NfL levels increase linearly across earlier disease stages.

Intriguingly, our ROC curve analysis showed a superiority of sNfL in discriminating HD mutations carriers from healthy controls compared to sGFAP. This is also in line with neuropathological studies in HD, which have shown that significant neuronal loss is observed at stage 0 of the disease, whereas astrogliosis first appears at stage 1 and continues to progress through to stage 4 [16].

We also conducted clinical biomarkers including cognitive tasks (TMT-A, TMT-B, SDMT) and olfactory testing. Our results showed significant differences in olfaction between all HD mutation carriers and HC, whereas the cognitive tasks (TMT-A, TMT-B, SDMT) were not able to detect differences between the preHD group and HC, suggesting that olfactory dysfunctions might be an even earlier non-motor symptom in HD than cognitive changes.

Mechanisms of olfactory dysfunction in HD discussed are pathological protein aggregation, neurodegeneration, or neuroinflammation [17, 18]. One neuropathological study showed mHtt aggregates in the olfactory bulbs of HD patients, similar to pathological aggregates of other neurodegenerative disorders [17].

According to our results, in premanifest and early disease stages, olfactory dysfunction and sNfL are able to detect subtle changes, whereas sGFAP and cognitive measurements show differences in more advanced disease stages.

## Conclusion

Taken together, in our study, we found that by using sNfL it is possible to detect changes in very early and even premanifest disease stages, whereas sGFAP may be more relevant in advanced disease stages.

Further studies with larger numbers of participants are warranted to assess the clinical utility of these clinical and body fluid biomarkers and to better classify subtle changes in very early disease stages.

## Data Availability

Anonymized summary data will be shared by reasonable formal request from qualified researchers if approved by the local Research Ethics Committee.
